# Rapid Detection of Porcine DNA in Meatball Using Recombinase Polymerase Amplification Couple with Lateral Flow Immunoassay for Halal Authentication

**DOI:** 10.3390/molecules27238122

**Published:** 2022-11-22

**Authors:** Mohd Hazim Mohd Yusop, Mohd Fadzelly Abu Bakar, Kamarul Rahim Kamarudin, Nur Fadhilah Khairil Mokhtar, Mohd Abd Motalib Hossain, Mohd Rafie Johan, Nor Qhairul Izzreen Mohd Noor

**Affiliations:** 1 Faculty of Applied Science and Technology, Universiti Tun Hussein Onn Malaysia, Hub Pendidikan Tinggi Pagoh, Muar 84600, Malaysia; 2 Faculty of Food Science and Nutrition, Universiti Malaysia Sabah, Jalan UMS, Kota Kinabalu 88400, Malaysia; 3 Halal Products Research Institute, Universiti Putra Malaysia, Putra Infoport, Serdang 43400, Malaysia; 4 Nanotechnology and Catalysis Research Centre, Institute for Advanced Studies, Universiti Malaya, Kuala Lumpur 50603, Malaysia

**Keywords:** porcine DNA, RPA, NALFIA, meatball, halal authentication

## Abstract

Point-of-care diagnostic methods for animal species determination are critical for rapid, simple, and accurate enforcement of food labelling. PCR is the most common method for species identification. However, the requirement of using a thermal cycler created drawbacks for the PCR application, particularly in low-resource settings. Hence, in this study, a method for porcine DNA detection using recombinase polymerase amplification (RPA), coupled with nucleic acid lateral flow immunoassay (NALFIA), was developed. Porcine-specific primers targeting pig (*Sus scrofa*) cytochrome b gene fragments specifically amplify a 197 bp fragment of the mitochondrial gene as being visualized by 2% agarose gel and PCRD NALFIA. The reaction temperature and time were 39 °C and 20 min, respectively. Herein, the specificity of the primers to porcine was confirmed after being assayed against six animal species, namely cow, goat, chicken, duck, dog, and rabbit. The porcine-specific RPA assay shows a high limit of detection of 0.01 ng/µL pork DNA. Based on the preliminary performance data obtained from this study, the potential of this method as a rapid and sensitive tool for porcine DNA detection in meat-based products is foreseen.

## 1. Introduction

Authentication of meat products by determining the origin of the animal species is important to detect adulteration practices and to protect consumers from fraudulence and deception. The reasons for meat adulteration happened because of economic profit [1,2] and untraceable cross-contamination via meat processing machinery [3]. Hence, a common situation of meat fraud because of economic profit would be either partially or entirely substitution of an expensive meat species with a similar or almost similar but cheaper one to reduce the cost of materials [4]. As for undetectable cross-contamination, it may happen when the same machinery used to process a different kind of meat species is improperly cleaned [5]. Other than that unintentional cross-contamination of halal meat products with pork or pork adulteration will refute the halal status of the meat [6]. In addition, given that there is high consumer demand for meat products, the increase in meat product prices also leads to meat adulteration [7].

Meatballs are a type of meat product from livestock that are very well known worldwide. They can be made from pork, chicken, beef, and fish meat, but beef meatballs are the most famous and widely available product on the market. Halal issues emerge if a product contains non-halal ingredients such as pork and its derivatives. This also can happen in meatball products sold in the market. Halal labels are sometimes applied to packaged meatballs to inform consumers of the halal status of the products. Although the meatballs sold in the market are mostly claimed to be beef meatballs, it is often found that the meat is adulterated using other non-halal meats. A study by Erwanto et al. (2014) used PCR-RFLP method and found nine cases of pork detected in beef meatball stalls around Yogyakarta Province in Indonesia. Thus, methods for meatball halal authentication are important to protect Muslim consumers from non-halal products [8].

Several methods have been developed for meatball authentication based on lipid [9], protein [10], and nucleic acid [11]. Simultaneously, the majority of the analytical tools that are usually used for meat authentication in meat products are molecular-based methods (Table 1). Lipid and protein-based analyses are usually not reliable because they are easily degraded during high pressures, heat treatments, and other processing techniques [12]. Therefore, both protein and lipid-based methods have been substituted by nucleic acids or molecular-based methods. Meanwhile, the supremacy of using molecular-based specifically DNA is because it is a stable molecule and heat-resistant [13]. Furthermore, a previous study reported successful DNA extraction and amplification from canned and highly processed meats [14].

**Table 1:** Application of DNA-based method for detection of animals’ source in meatballs.

Method	Target	Gene	Amplification Condition	Detection	Sensitivity	References
PCR	Pig, Monkey, Dog, Rat, Cat	ND5 ATPase Cytb	60–94 °C; 1 h	Electrophoresis	0.01–0.02 ng/µL	[15]
Real-time PCR	Dog	Cyt b	1 h	Fluorescence	0.5 pg/µL	[16]
Real-time PCR	Pig	d-loop	53–95 °C; 1 h	Fluorescence	5 pg/µL	[11]
Real-time PCR	Pig	Cytb d-loop	64–98 °C; 20 min	Fluorescence	0.01% binary meat mixture	[13]
Isothermal (LAMP)	Pig	d-loop	65 °C; 1 h	SYBR Green I	0.5 ng/µL	[17]
PCR	Cow, Buffalo, Chicken, Duck, Sheep, Goat, Pig	Cytb ND5	60–95 °C; 1 h	Electrophoresis	0.01–0.005 ng/µL	[18]
PCR	Dog, Pig, Monkey, Cat, Rat	ATPase ND5 Cytb	58–95 °C; 1.5 h	LFD	0.01–1 ppm	[19]
Isothermal (RPA)	Pig	Cytb	39 °C; 20 min	LFD	0.01 ng/µL	This work

In addition, using a species-specific primer, molecular methods such as polymerase chain reaction (PCR) have been widely used in porcine DNA detection in meatballs, including the PCR-RFLP [20,21], species-specific PCR [18] and real-time PCR [11,22]. However, there are some limitations to using PCR techniques. For instance, PCR requires an expensive thermocycler and takes a longer analysis time. Thus, the isothermal amplification method is now getting popular in replacing the PCR method. Meanwhile, analysis of porcine DNA in meatballs has been performed using loop-mediated isothermal amplification (LAMP) [21]. However, LAMP required six complicated designed primers while recombinase polymerase amplification only requires two common primers. On the contrary, RPA used a lower temperature for amplification of 39 °C, while the LAMP amplification temperature was 60 °C [23]. RPA assay for animal species has been developed for detection of sheep [24], duck [25], horse [26], and cow [27]. There are also RPA assays targeting pigs [26,28]. However, different detection of RPA products, such as SYBR green [28] and fluorescence [26], have been used. The detection of RPA products using NALFIA has advantages, such as being fast, sensitive, and low cost [29]. Meanwhile, there are limited studies on the application of isothermal amplification, particularly RPA, for the detection of porcine material in meatballs. Previous methods only used raw meats as samples and there is a lack of evidence for using RPA for processed meat products such as meatballs. Therefore, this study intended to design a specific porcine DNA primer targeting on mitochondrial cytochrome b (*cyt b*) gene and validating the recombinase polymerase amplification (RPA) method, coupled with nucleic acid lateral flow immunoassay (NALFIA) to produce a rapid DNA amplification result compared to the previous method.

## 2. Results and Discussion

### 2.1. Specificity of RPA Assays

**Figure 1:**
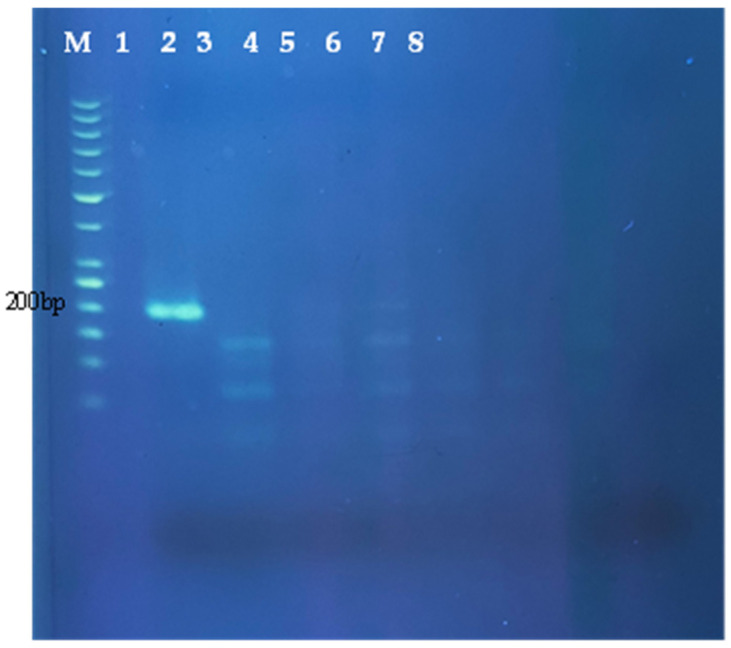
Electrophoresis profile of porcine-specific non-labelled primer RPA products amplified for pork. M—50 bp DNA ladder; 1—pork; 2—beef; 3—mutton; 4—dog; 5—chicken; 6—duck; 7—rabbit; 8—no template control (ntc).

Similar to PCR, RPA depends on primers to establish a sensitive assay. In general, the length of RPA primers is between 30–35 bases as compared to PCR, which is just around 20 bases. However, reports showed some PCR primers also worked for RPA assay [30]. The 197 bp porcine-specific cytochrome b region of pig mitochondrial DNA being chosen for RPA primers design has 100% interspecies sequence variability. There are two methods of amplicon detection employed in this study. The preliminary performance of the porcine-specific RPA assay was initially examined using non-labelled primers and the amplicon was visualized using 2% agarose gel electrophoresis. Moreover, improvement of the RPA amplicon visualization on the NALFIA device (PCRD) was then carried out to qualify the method as a point-of-care diagnostic method. In both amplicon visualization methods, the RPA assay conducted on the DNA of seven different animal species shows that the 197 bp amplicon was only obtained in a reaction containing pork DNA. Thus, the gel electrophoresis result showed a very clear single band obtained in a reaction containing pork DNA (Figure 1).

**Figure 2:**
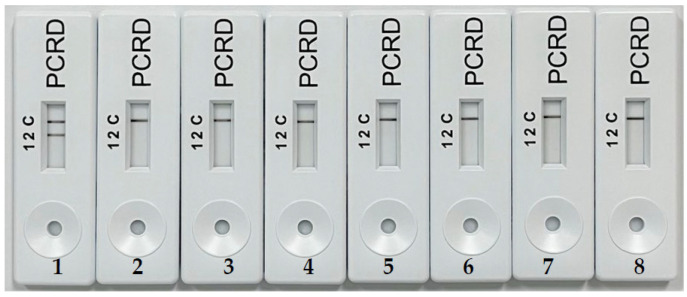
Specificity analysis of porcine DNA labelled primer by RPA with NALFIA; 1—pork; 2—beef; 3—mutton; 4—dog; 5—chicken; 6—duck; 7—rabbit; 8—no template control (ntc).

Meanwhile, two bands were visualized on PCRD of RPA amplicon generated using pork DNA. The reaction was carried out to test the specificity of the primers against all other six different types of meat samples, and nuclease-free water acted as no template control (NTC). Under the optimized RPA conditions, it has been demonstrated that only the RPA assay meant for visualization through PCRD employed a forward primer, being labelled with FAM, while the reverse primer was labelled with Biotin (Figure 2). Herein, the label is very important to make sure the RPA products can be detected by NALFIA. A specificity test showed that the NALFIA device named PCRD detected presence on porcine DNA by showing a clear test line (L2) and another clear control line (C) for assay control. This assay used antibodies against primer-tags (FAM and Biotin) and has been created for lateral flow detection of DNA amplification products from PCR or isothermal methods. The assay consisted of an absorbent pad, nitrocellulose membrane, conjugate pad, and a sample well, which are attached in a plastic cassette. Upon sample application, the carbon Biotin-conjugated antibodies were rehydrated and reacted with the tag/Biotin labelled amplicons in the sample. The first line (L1 not used in this study) consisted of anti-DIG monoclonal antibodies for capturing DIG/Biotin labelled amplicons. Meanwhile, the second line (L2) contains anti-FAM monoclonal antibodies to capture FAM/ Biotin labelled amplicons for porcine DNA assay. The excess carbons dots covered with Biotin antibodies flowed past the two test lines and were fixed at the reaction control line (C) to ensure that the test cassette operated correctly.

### 2.2. Sensitivity (Detection Limit)

**Figure 3:**
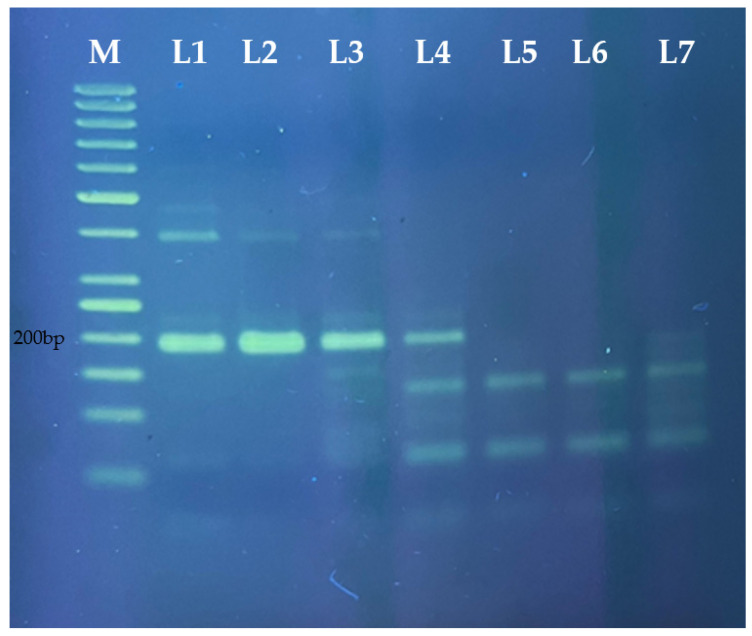
Electrophoresis profiles of RPA products amplified with porcine-specific RPA assay from the corresponding different amounts of DNA (M—50 bp DNA ladder, L1:10, L2:1, L3:0.1, L4:0.01, L5:0.001, and L6:0.0001) ng/µL with no template control (L7).

**Figure 4:**
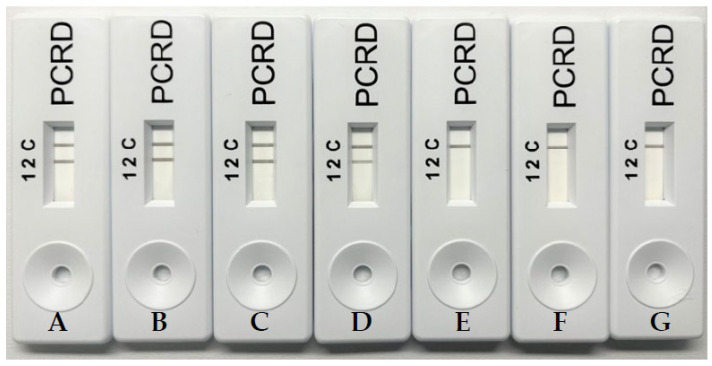
Sensitivity test of porcine DNA by RPA with NALFIA; ((**A**) 10, (**B**) 1, (**C**) 0.1, (**D**) 0.01, (**E**) 0.001, and (**F**) 0.0001) ng/µL with no template control (**G**).

**Figure 5:**
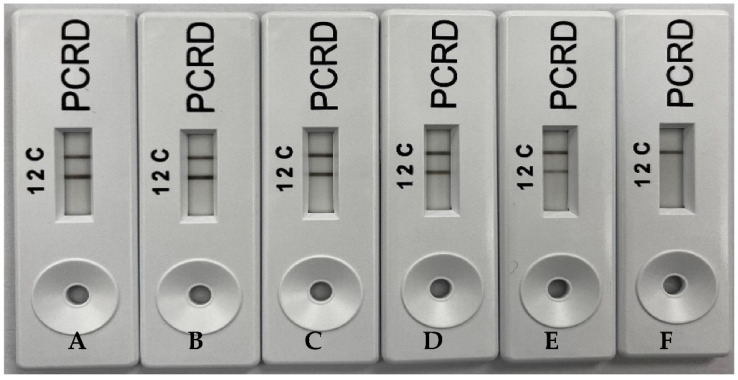
Sensitivity test of RPA-NALFIA assay for adulterated meatballs; ((**A**) 100%, (**B**) 50%, (**C**) 10%, (**D**) 1%, (**E**) 0.1%, and (**F**) 0% pork meatball in beef meatball).

Limit of detection of porcine-specific RPA assay for visualization using gel electrophoresis (Figure 3) and PCRD (Figure 4) was carried out using the 10-fold serial diluted pork. The detection limit for this porcine-specific RPA was found to be at 0.01 ng/µL. Ali et al. (2015) reported a detection limit of 0.02 ng/µL porcine DNA using real-time PCR. The RPA-NALFIA assay could also detect as low as 0.1% pork meatball in beef meatballs (Figure 5). Another study for porcine DNA detection using the isothermal method has also been done by Girish et al. (2020). They used the loop-mediated isothermal amplification (LAMP) method and the LOD was 0.5 ng/µL. On the one hand, this proves that this assay is more sensitive than the LAMP. On the other hand, RPA used simple designed primers with a minimum of two oligonucleotides in one reaction while LAMP used complicated designed primers with four or six oligonucleotides to run as compared to LAMP. Apart from that, the amplification temperature for LAMP is much higher (65 °C), while RPA requires a much lower reaction temperature (39 °C). Amplicon detection using NALFIA also increases the assay rapidity as compared to conventional agarose gel electrophoresis. Therefore, this assay could be conducted on simple equipment such as a water bath or thermoblock, and it does not need any thermal cycle equipment like PCR.

### 2.3. Application of RPA NALFIA in Meatball Samples

**Figure 6:**
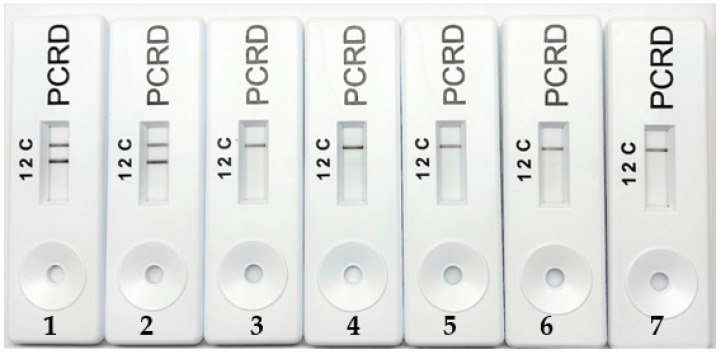
Analysis of RPA NALFIA on meatballs samples; (1: pork meatball brand A, 2: pork meatball brand B, 3: beef meatball brand C, 4: beef meatball brand D, 5: chicken meatball brand E, 6: chicken meatball brand F; 7: no template control).

The applicability of the porcine-specific RPA visualized on PCRD was then determined on commercial meatballs. The result in Figure 6 showed that among six samples, porcine DNA was only detected in pork meatballs using this method. There was no positive signal found in chicken and beef meatballs. Compared to previous methods shown in Table 1, this work showed the fastest time amplification reaction, which is 20 min. Amplification temperature in this work also recorded only 39 °C, the lowest temperature compared to PCR and LAMP method. Meanwhile, changing the non-labelled primers with the labelled ones means the assay can be detected easily and quickly by RPA NALFIA [24]. Furthermore, the RPA NALFIA method requires less hands-on time and is easy to perform, and the low demand for instruments like PCR thermal cycler would make the established method achieve a point-of-care testing in the future. Therefore, the RPA NALFIA has abroad application prospect in the authenticity detection of animal-derived food products [31].

## 3. Materials and Methods

### 3.1. Samples Collection and DNA Extraction

This study used raw samples from various types of meat, including pork, beef, chicken, duck, mutton, rabbit, and dog meat. It consisted of targeting and non-targeting various animal species to optimize and determine the specificity of primers, respectively. In addition, different kinds of meatballs were used, such as pork meatballs, chicken meatballs, and beef meatballs. All those samples were purchased from local supermarkets in Johor and Kuala Lumpur, Malaysia. Then, all the samples were stored at −20 °C in a freezer to prevent any unwanted enzymatic nucleic acid degradation of the samples before further continuing with the DNA extraction procedure.

Extraction of DNA from 2 g samples was done using a commercial genomic DNA extraction kit (Qiagen, Germany) based on the manufacturer’s protocol. The quantity and quality of isolated DNA were analyzed using a nanophotometer (Implen) at an absorbance of 260 nm and the quality or purity of DNA was checked based on the ratio A_260_/A_280_.

### 3.2. Design of Oligonucleotide Primers

Oligonucleotide primers for RPA used in this work were targeted to amplify a fragment of the porcine (*Sus scrofa*) cyt b gene. Gene sequence (Accession No. NC_002008.4) was obtained from the National Centre of Biotechnology Information http://www.ncbi.nlm.nih.gov (accessed on 3 January 2022). Primer3 Plus application software http://www.bioinformatics.nl/cgi-bin/primer3plus/primer3plus.cgi (accessed on 3 January 2022) was used for primer design. Then, the primers sequences were tested for homology with other species DNA sequences using the online Basic Local Alignment Algorithm Search Tool (BLAST). Moreover, all oligonucleotide primers were synthesized by Apical Scientific to be used for amplification of 197 bp DNA fragment of porcine cyt b gene (Table 2). For lateral flow immunoassay detection, the 5′ end of forward primer was labelled with Fluorescein (FAM) and the 5′ end of reverse primer was labelled with biotin.

### 3.3. RPA Assay for DNA Amplification

Herein, the RPA assay was performed using a TwistAmp^®^ Basic kit (TwistDx Limited, Cambridge, UK) for both labelled and non-labelled primers. Briefly, 1.5 μL of each primer (10 µM), 29.4 μL of rehydration buffer, and 14 μL of nuclease-free water were mixed thoroughly in the 0.2 mL tubes, followed by adding 2.5 μL of Magnesium acetate (280 mM). Afterwards, 1 μL of each nucleic acid template was added. All the tubes were centrifuged shortly before being put into the water bath at 39 °C for 20 min. Furthermore, a negative control was included in each run with nuclease-free water instead of a DNA template. Finally, the amplification reaction was observed after 20 min of incubation period using agarose gel (for non-labelled primers) and lateral flow assay (for labelled primers).

### 3.4. Gel Electrophoresis

In addition, RPA products from non-labelled primers were further analyzed using 2% agarose gel electrophoresis. The gel was prepared by dissolving a total of 3 g of agarose in 150 mL 1× Tris Borate EDTA (TBE) buffer pH 8.0. This solution was further heated until completely dissolved. Meanwhile, the electrophoresis was run at 120 V for 70 min and the RPA products were visualized using a gel documentation system (Cleaver Scientific).

### 3.5. Nucleic Acid Lateral Flow Immunoassay (NALFIA) Detection

Detection of RPA products using labelled primers was done using a lateral flow device named PCRD nucleic acid detector cassette (Abingdon Health Ltd., National Innovation Campus, Sand Hutton, York, UK). After the RPA reaction was completed, a total of 6 μL of the amplified product was transferred into a 0.2 mL tube and mixed thoroughly with 84 μL of the PCRD extraction buffer, then 75 μL of the mixture was added to the sample well of a PCRD test cassette. The cassette was placed in a horizontal position, and the results were recorded within 5 min.

### 3.6. Specificity and Sensitivity of the RPA Assays

Herein, the specificity of the RPA assays was analyzed using designed RPA primers against other animal species: cow, goat, chicken, duck, dog, and rabbit. Specificity tests were done for both labelled and non-labelled primers. In addition, the sensitivity of developed RPA assays was evaluated by using tenfold serially diluted genomic porcine DNA. Hence,10 ng to 0.0001 ng genomic porcine DNA dilutions were prepared using nuclease-free water and RPA assays were carried out using these diluted DNA solutions. The binary mixture containing 100, 50, 10, 1, 0.1, and 0 % (*w/w*) pork meatballs in beef meatballs were prepared and used. Then, RPA products were visualized by both gel electrophoresis and the NALFIA device.

## 4. Conclusions

The application and development of species-specific isothermal amplification method using RPA-NALFIA was found to be effective in detecting domestic meat species for human consumption. The presented work provided proves that the use of the porcine-specific labelled primer pair designed, coupled with the optimized RPA-NALFIA method, is a fast, reliable, specific, and sensitive method. Hence, this is also an essential discovery for halal authentication in food products. In summary, this RPA-NALFIA method could be really applied to halal meat authenticity detection in the future.

## Figures and Tables

**Table 2:** Oligonucleotide sequences of the primers used in this study.

Primers	Length	Sequences	Description
SSF3	33	5′-CACTATTAAAGACATT CTAGGAGCCTTATTTAT-3′	Porcine Non-Labelled Forward Primer
SSR3	33	5′-CACCTAGTTTATTAGGAATTGAACGTAGAATAG-3′	Porcine Non-Labelled Reverse Primer
SF2F	33	5′FAM-CACTATTAAAGACATT CTAGGAGCCTTATTTAT-3′	Porcine Labelled Forward Primer
SR2B	33	5′Biotin-CACCTAGTTTATTAGGAATTGAACGTAGAATAG-3′	Porcine Labelled Reverse Primer

## Data Availability

Not applicable.
